# Particles’ Organization in Direct Oil-in-Water and Reverse Water-in-Oil Pickering Emulsions

**DOI:** 10.3390/nano13030371

**Published:** 2023-01-17

**Authors:** Diego M. Ramos, Véronique Sadtler, Philippe Marchal, Cécile Lemaitre, Frédérick Niepceron, Lazhar Benyahia, Thibault Roques-Carmes

**Affiliations:** 1 Laboratoire Réactions et Génie des Procédés (LRGP), UMR 7274 CNRS, Université de Lorraine, 1 Rue Grandville, 54001 Nancy, France; 2 Institut des Molécules et Matériaux du Mans (IMMM), UMR 6283 CNRS, Le Mans Université, 1 Avenue Olivier Messiaen, CEDEX 9, 72085 Le Mans, France

**Keywords:** Pickering emulsions, dodecane, paraffin, silica, reverse emulsions, direct emulsions

## Abstract

This paper addresses the impact of the particle initial wetting and the viscosity of the oil phase on the structure and rheological properties of direct (Oil/Water) and reverse (Water/Oil) Pickering emulsions. The emulsion structure was investigated via confocal microscopy and static light scattering. The flow and viscoelastic properties were probed by a stress-controlled rheometer. Partially hydrophobic silica particles have been employed at 1 and 4 wt.% to stabilize dodecane or paraffin-based emulsions at 20 vol.% of the dispersed phase. W/O emulsions were obtained when the particles were dispersed in the oily phase while O/W emulsions were prepared when the silica was introduced in the aqueous phase. We demonstrated that, although the particles adsorbed at the droplets interfaces for all the emulsions, their organization, the emulsion structure and their rheological properties depend in which phase they were previously dispersed in. We discuss these features as a function of the particle concentration and the oil viscosity.

## 1. Introduction

The use of particles inside dispersed liquid/liquid systems is a relevant option to stabilize emulsions [1,2]. These peculiar types of emulsions are denoted as Pickering emulsions. It is generally admitted that the presence of the particles hinders significantly the coalescence of the droplets [3]. In addition, the recent uses of bioparticles allow the development of innovative products based on Pickering emulsions [4,5,6].

The type of emulsions, direct oil-in-water O/W and reverse water-in-oil W/O, depends on the oil-versus-water wetting preference. The wettability is characterized by the contact angle of the particles at the water/oil interface [7]. The first approach is empirical and considers that the phase which preferentially wets the particles is the continuous phase of the emulsion [8]. Another approach considers that the initial dispersion of the particles (in oil or water) during the preparation of the emulsions influences also the type of emulsions. In the case of intermediate wettability, the continuous phase of the emulsion corresponds to that in which the particles are initially dispersed [9,10]. The origin of this versatility comes from the contact angle hysteresis [11]. Actually, for particles inside the water, the receding contact angle has to be used, while for particles in oil, the advancing contact angle has to be analyzed. The other explanation comes from surface heterogeneities on the particles surface, in particular its roughness [12]. In fact, the surrounding initial liquid around the particles (initially trapped by the surface roughness) has a non-neglectable influence on the mobility and adsorption of the particles at the interface. Indeed, trapped liquid on particle roughness controls the desorption energy of the particle from the W/O interface [13]. For an optimal anchoring at the interface, the initial film of liquid on the particles has to dewet from the surface of the particles.

The repartition and organization of the particles at the liquid/liquid interface and in the continuous phase is an important parameter defining end-user properties [14]. They depend on several parameters such as the contact angle, the wettability, and also the phase in which the particles are initially dispersed [7,12]. Several configurations have been reported. In some particular cases, the majority or all the particles are anchored at the interface of the droplets [7]. On the opposite, in the other extreme configuration, the majority of the particles is in the continuous phase [15]. The organization of particles and dispersed phase in Pickering emulsions could be controlled by the external (continuous) phase of emulsion. More precisely, hydrophobic (i.e., induced dipoles) or hydrophilic (i.e., hydrogen bonds or permanent dipoles) interactions between the stabilizing particles and the continuous phase can create droplet–particle networks into Pickering emulsions [16]. Barros et al. obtained a network of hydrophobic silica that interconnected droplets of silicon oil dispersed in water [17]. Velandia and coworkers demonstrated, through rheological characterizations, the existence of a tridimensional network of particles linking droplets of water in dodecane [18]. Ganley and van Duijneveldt obtained hexadecane-in-water emulsions stabilized by a network of montmorillonite platelets [19]. Dinkerve and colleagues also observed the formation of a clay particle network with confocal microscopy when stabilizing silicon oil-in-water emulsions [15]. While the influence of the viscosity or the ratio of viscosities of oil and water is well known on the droplet size distribution [10,20,21,22,23], its impact on the organization of the particles in Pickering emulsions has been less studied.

In this paper, some parameters that can impact the organization of the particles in Pickering emulsions are addressed. The influence of the liquid polarity, more precisely the phase in which the particles are initially wetted prior to the emulsification, and the viscosity of the oily phase on the organization of the particles in the emulsions, is particularly studied. To this aim, a content of 1 or 4 wt.% of the same silica particles was dispersed in the oily or aqueous phase to stabilize, respectively, reverse W/O or direct O/W Pickering emulsions, with 20 vol.% of the dispersed phase. The high concentration of silica of a 4 wt.% was used to enhance the possible effect of particles in the continuous phase (such as gel for instance). Dodecane and paraffin oils were used, since they have about the same physico-chemical nature, being mineral oils while displaying large differences in viscosity, i.e., *η_Paraffin/_η_Dodecane_* ~ 100. The effect of polarity was studied by comparing water and dodecane in order to neglect the impact of the viscosity, since *η_Water_* ~ *η_Dodecane_*. Paraffin oil was also used to evaluate the impact of the viscosity of oils by comparing dodecane/water and paraffin/water emulsions as well as water/dodecane and water/paraffin emulsions.

## 2. Materials and Methods

### 2.1. Materials

Paraffin oil was provided by Fisher Scientific; it was declared as general purpose grade. The n-dodecane was provided by Honeywell (99% of purity). All the aqueous phases corresponded to a brine of NaCl at 2 wt.% with deionized water (Milli-Q, 18 MΩ·cm). NaCl was provided by Sigma-Aldrich (St. Louis, MO, USA) (purity of 99.5%). Partially hydrophobic silica particles HDK H30 from Wacker Chemie AG were employed as stabilizing agents. The particle size of the dry native particle was declared to be equal to 20 nm from supplier technical data. Rhodamine B and Nile red were provided by Sigma-Aldrich Chemie GmbH.

### 2.2. Characterization of Partially Hydrophobic Silica Particles

The characterization of the hydrophobicity degree of the partially hydrophobic silica should help to understand the versatile behavior of these particles. The characterization of their wettability, or their wetting preference, may shed light on their behavior in reverse and in direct emulsions. To do so, the contact angle and the critical surface energy of the particles were measured.

#### 2.2.1. Contact Angle Measurement

Measurement of the triphasic contact angle was made in two steps: fabrication of a silica tablet and measurement of the contact angle.

**Figure 1:**
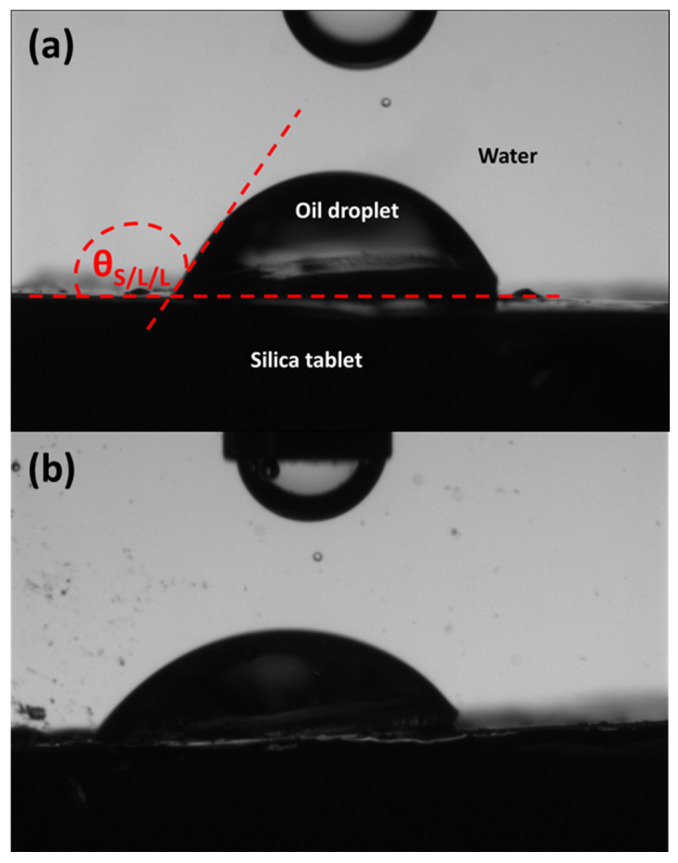
Measurement of the solid/liquid/liquid contact angle (θ_S/L/L_) of (**a**) silica/water/dodecane, and (**b**) silica/water/paraffin.

A tablet of silica particles was made through a compression process in a hydraulic press from Instron equipped with a 50 kN force sensor using a protocol developed in our laboratory [24]. The diameters of the matrix and of the punch were, respectively, 20 mm and 19 mm. The maximal compression force was fixed to 10 kN. To make the tablet and avoid cohesion with the work plan of the press, a small sheet of polypropylene was placed between the work plan and the matrix. Then, the matrix was filled with the silica particles, which were compressed a first time. After that, the matrix was filled with silica particles one more time and another compression cycle was carried out. The compression cycle was repeated 3 times. At the end, a bluish tablet of silica particles was obtained. The triphasic contact angle was measured with the fabricated tablet of silica particles. It was measured with the optical contact angle-measuring system OCA 15EC^®^. At the beginning, the silica tablet was introduced in the bottom of the glass cell. Then, the cell was filled with water. The needle containing the oil was introduced in the glass cell in the vicinity of the silica tablet. Finally, an oil droplet of 1–5 µm of diameter was dropped off under the tablet and the triphasic solid/liquid/liquid (θ_S/L/L_) contact angle was measured (Figure 1).

#### 2.2.2. Critical Surface Energy Measurement

To determine the critical surface energy of the silica particles, they were sprinkled onto the surface of a liquid. The liquid consisted of mixtures of water/propan-2-ol. The mass fraction of alcohol was varied between 10 and 20 wt.% which corresponded to liquid/vapor surface tension ranging between 41 and 31 mJ m^−2^. The critical surface energy of the particles was determined as the value of the liquid/vapor surface tension corresponding to the mixture containing the minimum amount of propan-2-ol, at which the particles sunk into the liquid [25,26].

### 2.3. Preparation of Silica Particle Suspensions

Two kinds of suspensions were prepared prior to emulsification. The particles were dispersed in the oil phase (oily suspension) or in the aqueous phase (aqueous suspension). The only difference in the protocol was the time of stirring which was significantly longer for the aqueous dispersion. In detail, a predefined quantity of silica, according to the desired formulation, was poured into a 56 mL of oily phase (or aqueous phase). First, two fractions of silica of 1 and 4 wt.% relative to the oil volume (or water volume) were prepared, corresponding to a mass of silica of 0.56 g and 2.24 g, respectively. Second, the silica wetting process by the oil was facilitated by means of a magnetic stirrer for 10 min. In the special case of the aqueous dispersion, this step lasted at least 48 h, until no more particles were observed at the surface of the suspension. This was due to the hydrophobic character of the silica particles. Third, the wetted silica was dispersed into the lipophilic phase (or hydrophilic phase) with the 550 Fisher sonic probe for 10 min at 120 W of power and ultrasonic pulsations of 2 s.

### 2.4. Preparation of Direct and Reverse Emulsions

All the emulsions were prepared with a dispersed phase fraction of 20 vol.% unless stated otherwise. The aqueous phase for all emulsions was a brine of 2 wt.% NaCl prepared with deionized water. The rotor-stator was an UltraTurrax^®^ DI 25 Basic and the S 25 N-10G stem from IKA-Werke GmbH & CO.

The reverse W/O and direct O/W emulsions were prepared in a batch mode. A volume of 14 mL of the dispersed phase (aqueous or oily phase) was poured into the previously prepared silica suspension in oil (dispersion of silica in dodecane or paraffin) or in brine. A clearly separated biphasic system was obtained in all cases. After that, an equivalent length of 2 diameters of the rotor-stator stem was immersed into the continuous phase (oily or aqueous phase). Then, the rotor-stator was turned on at 13,500 rpm for 10 min. A total emulsion volume of 70 mL was prepared.

### 2.5. Characterization of the Emulsions

Rheological behavior and the droplet size distribution of the Pickering emulsions as well as the droplet and particle organization into them were performed. The rheological characterization method was independent of the emulsions’ nature (W/O or O/W). However, the presence of a continuous oily phase in the emulsions forced the use of different protocols to measure droplet size. Due to the initial dispersion of silica particles in aqueous or oily medium, protocols of fluorescent labeling of the particles in emulsions were different even if images were obtained with the same confocal microscope. Dilution experiments and conductivity measurements were constantly performed in order to verify the type of emulsion (W/O or O/W).

#### 2.5.1. Rheological Characterization

The rheological behavior of the suspensions and the emulsions was characterized with an ARES strain-controlled rheometer from TA Instruments. All the tests were made at 20 °C; the temperature was controlled by a Peltier system. A plate–plate geometry of 50 mm in diameter was chosen for all the tests. The gap was fixed at 1 mm. First, the emulsion was gently shaken. After that, a sample of emulsion was placed over the lower plate with a spatula and the upper plate was then lowered until a gap of 1 mm formed. The sample volume was enough to fill the gap. Then, the rheological tests were made in the following order for a same sample of emulsion: flow test (steady-state regime), strain sweep test (oscillatory regime) and frequency sweep test (linear oscillatory regime). Each rheological test was repeated three times. The main parameters of the flow test were a shear rate sweep between 100 s^−1^ and 1 s^−1^ in logarithmic sweeping mode, including, for each shear rate, a delay before measure fixed at 20 s to ensure a steady-state regime achievement followed by a measurement averaged during 20 s. To determine the linear viscoelastic domain of the samples, strain sweep tests were made for deformations between 0.01 and 1 at a constant frequency of 10 rad/s in a logarithmic sweeping mode. Frequency sweep tests were performed at a constant strain of 0.01 between 100 rad/s and 1 rad/s in a logarithmic sweeping mode.

#### 2.5.2. Droplets Size Distribution

For reverse W/O emulsions, the droplet size distribution was obtained from snapshot captures and image analysis. The images were taken with a digital microscope: DinoLite AM4515T8. The magnification was fixed at ×900. The images were analyzed with the software Fiji from ImageJ.

For direct O/W emulsions, the droplet size distribution and the average droplet size of the emulsion were measured with a MasterSizer 2000^®^. This equipment used a static light-scattering technique combined with the Fraunhoffer diffraction model to obtain the droplet size distribution of the emulsion. The concordance of the size obtained with the two methods, i.e., granulometric measurements and microcopy, was verified for both direct and reverse emulsions.

#### 2.5.3. Fluorescent Confocal Microscopy

Confocal microscopy images were obtained with a confocal microscope, LSM 800, from ZEISS International. Fresh silica suspensions and Pickering emulsions were prepared on purpose to obtain images from confocal microscopy.

For fluorescent labeling in reverse W/O emulsions, the particles were labeled before emulsification (into suspensions) as follows. A mother solution of 500 mg/L of Nile red was prepared (in dodecane and in paraffin oil) and then diluted in order to have a final concentration of 10 ppm into different oily particle suspensions and reverse Pickering emulsions. They were stocked in a dark fresh place. The mother solution and samples were red–purple colored.

For fluorescent labeling in direct O/W emulsions, particles were labeled before emulsification as follows. A mother solution of 100 mg/L of rhodamine B was prepared and then diluted in order to have a final concentration of 10 ppm into different particles suspensions and emulsions. They were stocked in a dark fresh place. The mother solution and samples were pink colored.

## 3. Results and Discussions

### 3.1. Behavior of the Silica Particles at the Oil/Water Interface

The characterization of the particles was performed in terms of wettability and sprinkling. Figure 1a corresponds to the picture of a dodecane droplet on a tablet of compressed silica particles where the solid/liquid/liquid angle is indicated. The hydrophobic silica has a contact angle of 122°. When trying to measure the contact angle of water droplet in the presence of dodecane around the tablet, the measurement was not possible due to the redispersion of the silica particles in dodecane. This corresponds to the disaggregation of the tablet of silica. In other words, this might correspond to a total wetting of the dodecane onto the silica, i.e., the contact angle is equal to 0°. This result highlights the existence of a contact angle hysteresis, which is expected to favor the versatility of the particles to stabilize both direct and reverse emulsions. In the literature, it is claimed that the water/oil/particles contact angle drives the type of emulsion, and it can be linked to the wettability of the silica particles [11,27,28,29]. Particles having a contact angle larger than 90° should stabilize reverse W/O; otherwise, direct O/W emulsions are expected. However, this is not always the case since, here, the contact angle remains constant while the type of emulsion changes, as described in Section 3.3 and Section 3.4. Indeed, reverse emulsions are obtained only when the particles are initially wetted by the oil. When the particles are dispersed in the water, despite the contact angle value being higher than 90° and the large silica aggregates, direct O/W emulsions can be prepared. These results correspond well with findings of the literature [9,10,11]. The solid/liquid/liquid contact angle becomes equal to 130° ± 8.0° when the dodecane droplet is replaced by a droplet of paraffin (Figure 1b). This value is relatively close to that obtained with dodecane. Based on the uncertainty of the measurement, mainly due to the possible roughness of the silica tablet [24], it is considered that the contact angle values are similar for dodecane/water/silica and paraffin/water/silica. This confirms the similar chemical nature of the two oils.

The technical data sheet of the HDK H30 silica particles indicated that the half of the silanol groups of the surface were functionalized with dichloromethylsilane, which would correspond to one group of silanol (SiOH) per nm^2^ [29]. Binks and Clint considered that the percentage of silanol groups remaining on the surface of modified silica particles could be linked to their degree of hydrophobic behavior and, hence, to the behavior of the liquid/liquid/solid contact angle [30]. Those works presented the behavior of the contact angle of silica particles with different percentages of remaining silanol groups at a water/oil interface for different oils. For the sake of better understanding of our work, we paid focus to the system where a silica particle has 50% of remaining silanol groups and stabilizes a dodecane-in-water emulsion. In this case, the contact angle was estimated to be 80.5°. However, our system showed a value of 122° that would correspond rather to a remaining percentage of silanol on the surface of the silica particle under 25% [30]. This suggests a rather hydrophobic nature of the HDK H30 silica particles.

Furthermore, when measuring the critical interfacial tension of these silica particles, they remained on the liquid surface for a mass fraction of 10 wt.% of propan-2-ol. Conversely, when the mass fractions were larger than or equal to 12 wt.%, the particles had sunk into the mixture. Consequently, the critical surface energy of silica particles was equal to 38 mJ m^−2^. This value was also rather consistent with hydrophobic silica particles described in the literature [24,31]. In parallel, similar experiments were conducted with water, paraffin and dodecane liquids. When the silica particles were deposited onto the water, the particles remained at the surface. On the other hand, the particles were totally incorporated into the dodecane and the paraffin. In addition, it is important to note the larger duration time of stirring for the preparation of the aqueous dispersion (48 h) in comparison to that of the oil one (10 min). This also confirms the hydrophobic nature of the silica particles and corresponds well with the previous results for which the silica was sprinkled onto the different liquid interfaces.

These first sets of experiments suggest that these silica particles behave rather as hydrophobic particles, so they would be more likely to stabilize W/O emulsions. As they do not have the same affinity for the polar and non-polar phases, it would be interesting to know if they behave in a different way when dispersed in oil and when dispersed in water. The affinity of SiO_2_ for dodecane and paraffin also appears rather close.

### 3.2. Organization of Partially Hydrophobic Silica Particles in Oily or Aqueous Suspensions

**Figure 2:**
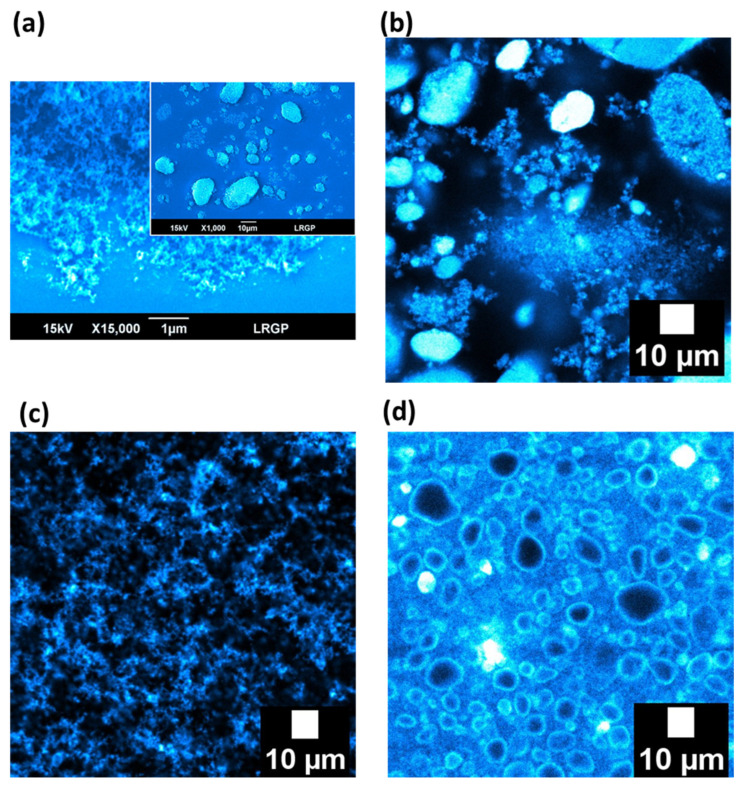
Scanning electron microscopy of (**a**) dry silica particles. Confocal fluorescence images of suspensions of silica particles in (**b**) dodecane, (**c**) paraffin oil and (**d**) water. The concentration of silica particles is 4 wt.%. Particles are represented in blue.

Figure 2a displays the dry silica powder. Large (~10–20 µm) and small (<1 µm) aggregates of silica were observed. The particles can be dispersed in oils as well as in water indicating that the silica has an “amphiphilic” behavior. Figure 2b,c show the organization of the partially hydrophobic silica particles when dispersed in an oily phase (dodecane or paraffin oil, respectively). In paraffin, the dispersion of the particles is the most homogeneous. This allows a better interconnectivity among particles, compared to the suspensions in dodecane or water, where interconnectivity is less obvious. Large aggregates of silica are still present in dodecane (Figure 2b). When dispersed in water, the majority of the silica particles remain aggregated (Figure 2d), similar to in the solid dry state. The silica particles form big micrometric aggregates, corresponding to the non-spherical dark objects with a blue corona or being completely blue.

**Figure 3:**
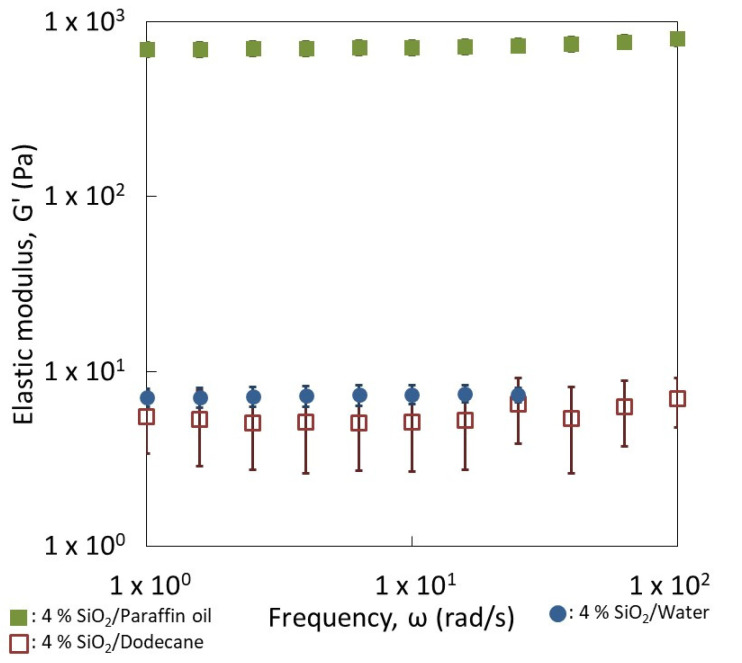
Rheological behavior of oily (dodecane and paraffin oil) and aqueous suspensions prepared with 4 wt.% of silica particles. Mechanical spectra showing the elastic modulus (G′) as a function of frequency (ω).

To confirm the interconnectivity between the particles, Figure 3 compares the elastic moduli of silica particle suspensions (4 wt.% of silica) prepared in dodecane, paraffin oil and water. Figures S1–S3 of the Supporting Information display the evolution of the elastic G′ and viscous G″ modulus as a function of the oscillatory frequency of those suspensions of silica particles. The elastic modulus G′ is larger than the viscous modulus for all the strains and frequencies evaluated. In addition, the elastic modulus remains constant with the frequency, which is indicative of a solid-like viscoelastic behavior. These three behaviors reflect the existence of a three-dimensional connectivity of silica particles that extends throughout the volume of the samples. We also see a break of this network, characterized by a drop in the two moduli with a crossing point, beyond which G″ becomes greater than G′ with the passage of G″ through a maximum (around strain = 0.3) which characterizes the dissipation of energy during the break in the network (Figures S4 and S5 of the Supporting Information, G′, G″ vs. strain). This is the so-called Payne effect. The cross-over point G′/G″ corresponds classically to the yield stress, which is the minimum stress that must be applied to make the material flow and, thus, cause the network to break. The rupture demonstrates the formation of a physical gel which is also reversible.

Consequently, these partially hydrophobic silica particles are able to interact independently of the medium, even if the nature of interactions is not the same. An attractive network of particles is expected in paraffin and dodecane (Figure 2b,c) while repulsive forces between the silica particles are expected in water (Figure 2d). However, the strength of networks is not the same. Indeed, the silica suspension in paraffin behaves as the strongest gel with an elastic modulus G′ of 600–700 Pa. In contrast, the strength of the gel formed into dodecane or water appears much lower than that obtained in paraffin. Their elastic moduli are about 7 Pa in the linearity region. It seems appropriate to speak about weak gels of silica in dodecane or water. The network of silica is less strong in those cases when compared to the silica particles dispersed in paraffin oil. Indeed, the biggest interconnectivity of particles was observed in the paraffin oil (Figure 2c). The strongest gel was obtained with the most viscous liquid.

### 3.3. Reverse and Direct Emulsions with Liquid Phases of Close Viscosity Values (η_Dodecane_ ≈ η_Water_)

In this part, water and dodecane were used in order to neglect the impact of the viscosity. Here, the effect of the liquid polarity or the impact of the initial wetting of the particles in oil or in the aqueous phase prior to emulsification on the organization of the particles is addressed. Water/dodecane and dodecane/water emulsions were obtained depending on the initial wetting of the particles. When the particles were initially dispersed in water, dodecane/W emulsions were obtained since the emulsions can be diluted in water and have a conductivity values around 800–1000 µS cm^−1^. When the silica was initially wetted by the oily phase, W/dodecane emulsions are reported regardless of the silica content (1–4 wt.%). They present the ability to be diluted in dodecane and exhibited conductivity values lower than 0.1 µS cm^−1^, which confirms the presence of a reverse W/O emulsion.

**Figure 4:**
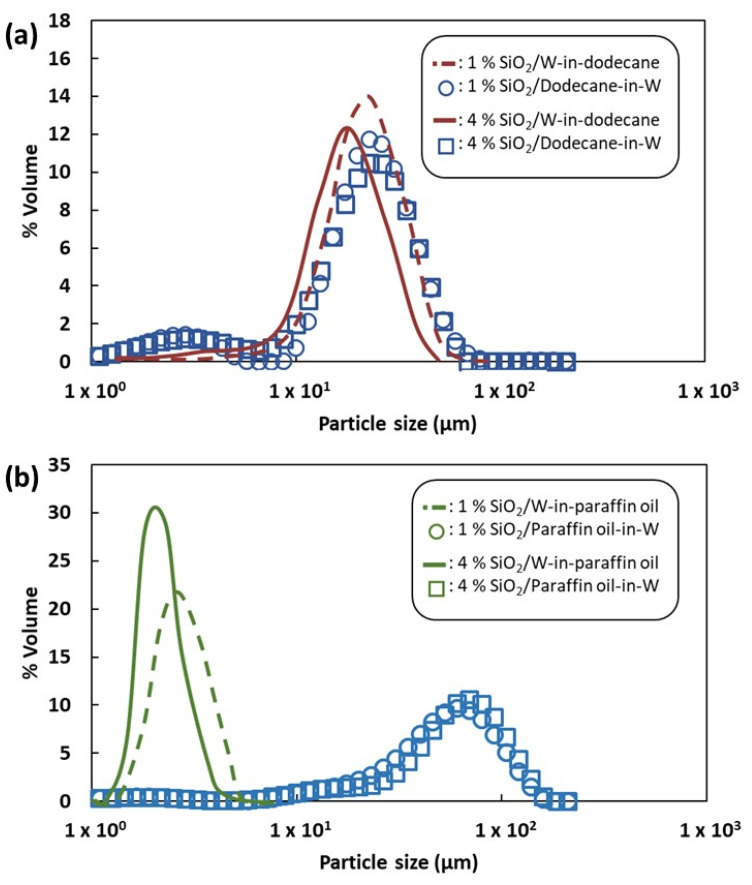
Droplet size distributions of (**a**) dodecane: reverse water-in-dodecane and direct dodecane-in-water emulsions, and (**b**) paraffin: reverse water-in-paraffin oil and direct paraffin oil-in-water emulsions with 1 or 4 wt.% of silica particles.

Table 1 shows the diameters of reverse and direct emulsions prepared at two different contents of silica particles, with dodecane and water. Their droplet size distributions are shown in Figure 4a,b. The droplet diameters of emulsions prepared with different continuous phases seem to remain unchanged. This could be explained by the viscosities of both liquid phases, which are very close ($\eta_{Dodecane}$ = 1.4 × 10^−3^ Pa s and $\eta_{Water}$ = 1.0 × 10^−3^ Pa s). Indeed, shearing efficiency of the dispersed phase by the emulsification device increases with the viscosity of the continuous phase, which means that the average droplet size diminishes [11]. If there is no significant difference between viscosities of both phases, the same droplet size could be expected for reverse and direct emulsions. This is true also because the particles are able to stabilize the two types of emulsions (O/W and W/O).

**Figure 5:**
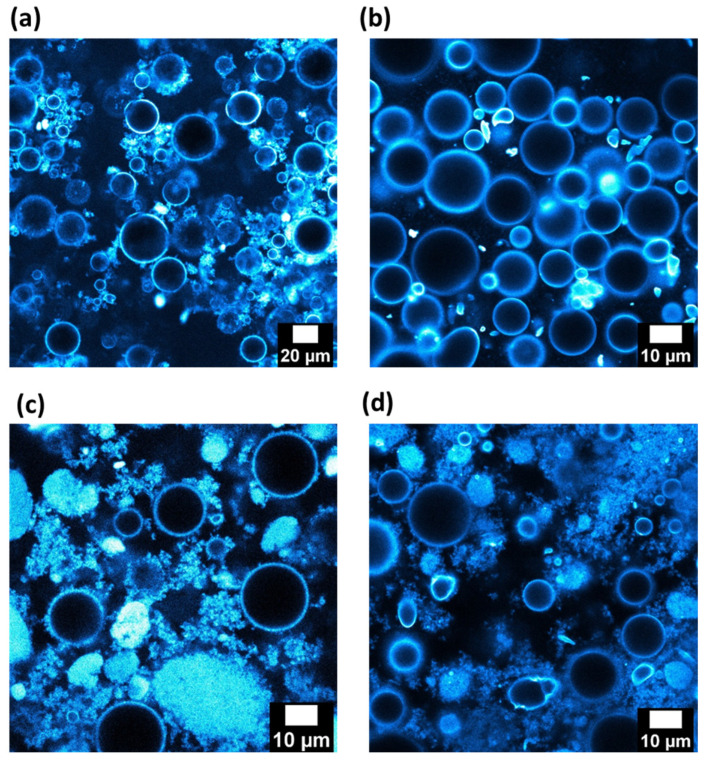
Confocal fluorescence images of (**a**,**c**) reverse Water/Dodecane and (**b**,**d**) direct Dodecane/Water emulsions prepared with (**a**,**b**) 1 wt.% and (**c**,**d**) 4 wt.% of silica particles.

Images, obtained by confocal microscopy, of reverse and direct emulsions stabilized with 1 wt.% of silica in the continuous phase are shown in Figure 5a,b. The silica particles are represented in a bluish coloration and the dispersed phases correspond to black round objects in each image. Both kinds of emulsion have populations of droplets with a similar size, as shown before in Table 1. It can be considered that the liquid interfaces are covered with the silica particles, as the droplets have a bluish corona regardless of the nature of the emulsion. This confirms the adsorption of the particles at the dodecane/water interfaces independently of the initial phase in which the particles are introduced. The behavior of the aggregates of silica is not the same following the nature of the continuous phase. Indeed, when it is dodecane (Figure 5a), particles of the partially hydrophobic silica remain in the continuous phase and seem to create interconnections among droplets. In the aqueous continuous phase, the majority of the silica particles diffuse towards the oil/water interfaces (Figure 5b).

**Figure 6:**
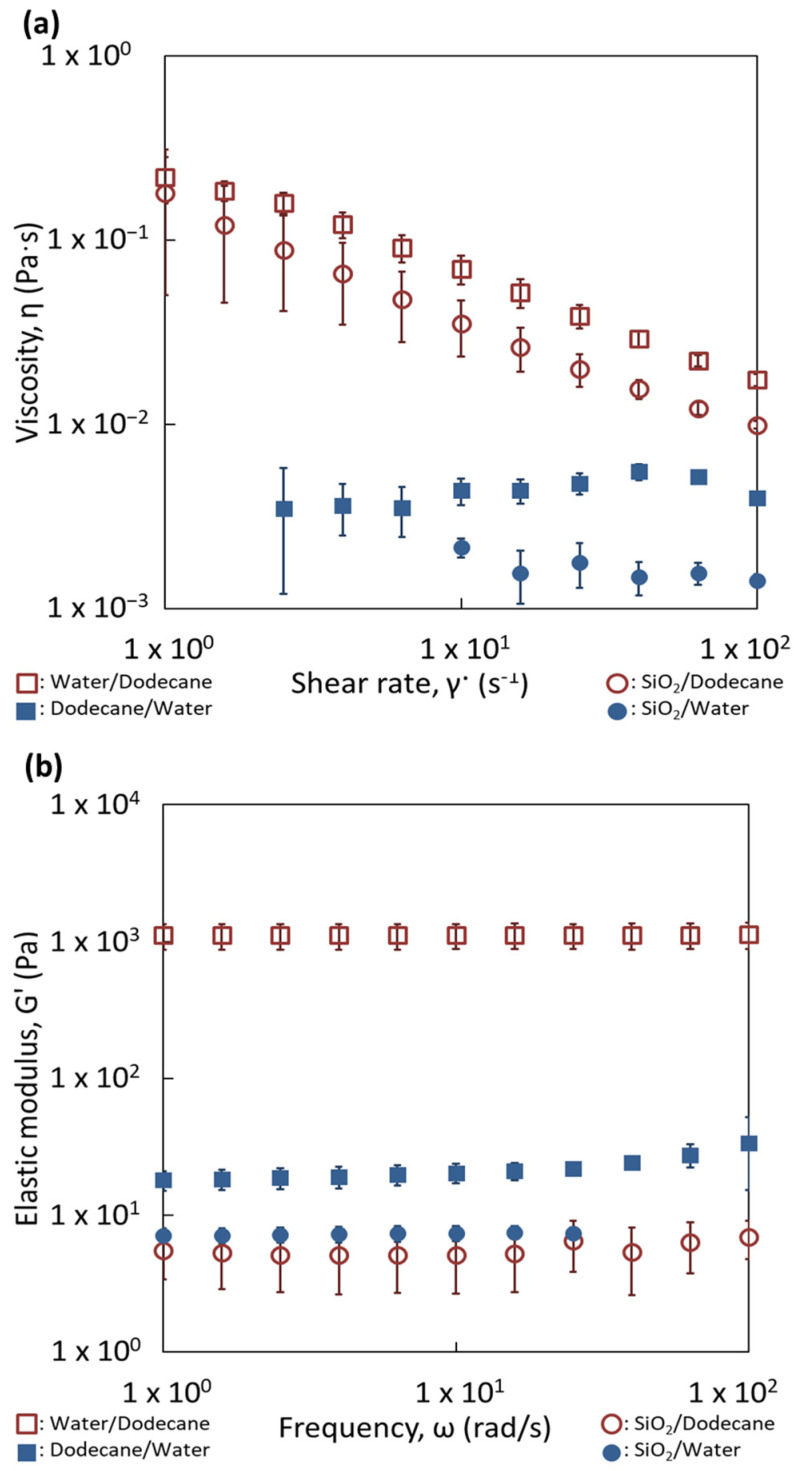
Comparison of the rheological behavior of reverse and direct emulsions prepared with dodecane with (**a**) 1 wt.% and (**b**) 4 wt.% of partially hydrophobic silica particles. (**a**) Flow curves. (**b**) Mechanical spectra.

The behavior of viscosity against the shear rate of oily and aqueous suspensions with a content of 1 wt.% of silica particles are represented in Figure 6a. The viscosity of the oily suspension exhibits a non-Newtonian behavior. Its viscosity is significantly higher than that of the aqueous suspension. The viscosity difference can be attributed to the different nature of inter particle interactions, leading in particular to compact aggregates in the aqueous suspension (Figure 2b) while it forms more open and branched aggregates in dodecane (Figure 2d). The viscosity behavior of the reverse W/O emulsion exhibits also a non-Newtonian behavior. The viscosity values are larger than those of the suspension of silica in dodecane. The viscosity of the reverse emulsion is also higher than the O/W direct emulsion. Since the viscosities of both liquid phases at their pure state are very close and since they increase when silica particles are dispersed, the difference in viscosity between both emulsions can be attributed mainly to the interactions created among silica particles in the continuous oily phase (Figure 5a,b). Consequently, the rheological behavior is dominated by the dispersion of silica in the continuous phase, since the emulsions follow the same evolution as the suspensions. The presence of droplets does not seem to be the predominant factor in the rheological behavior, although it is possible that some connectivity between droplets is favored by the silica particles (Figure 5a) leading to a slight increase of the viscosity of the emulsions compared to that of the silica suspensions. To confirm this aspect, a larger amount of silica was used (4 wt.%).

Emulsions of dodecane and water were also prepared with 4 wt.% of partially hydrophobic silica in the continuous phase. The droplet size does not significantly vary when the silica amount is shifted from 1 wt.% to 4 wt.%. Images from these emulsions were obtained by fluorescent confocal microscopy (Figure 5c,d). Droplet size distributions of both emulsions seem to be the same, as expected from Table 1. The bluish corona around the droplets of reverse and direct emulsions corresponds to the presence of particles of silica on the liquid droplets. This phenomenon was already reported with a lower amount of silica. However, in comparison with dodecane and water emulsions prepared with 1 wt.% of silica particles (Figure 5a,b), the presence of more silica in the continuous phase has to be emphasized. This is logical since the content of silica was increased by four-fold, i.e., from 1 to 4 wt.%. This remaining silica seems to create a network in both cases. In addition, particles aggregates were observed in the continuous phase, as was the case for the particles suspensions in dodecane or water (respectively, Figure 2b,d).

In order to quantify the strength and number of contacts of each network, the elastic moduli (G′) of both emulsions were measured as a function of the frequency (Figure 6b). In both cases, the values of the elastic moduli were independent of the frequency applied and higher than the viscous moduli, which is the distinctive behavior of a gel. This is surprising for 20 vol.% of dispersed phase since the gel behavior of classical surfactant emulsions takes place for contents of dispersed phases larger than 50% [32,33,34,35,36,37,38]. This could mean that percolation (contact between droplets) in our case is mainly owing to the presence of the silica aggregates in the continuous phase. However, the strength of both networks is not the same, since the values of G′ are quite different (1000 Pa vs. 20 Pa). The reverse emulsion has the strongest one. The incomplete connectivity observed for the silica particles in dodecane (Figure 2b) is compensated by the water droplets, resulting in a network producing higher elastic moduli. Indeed, it can appear surprising to obtain a gel of silica with this relatively low amount of silica, i.e., 4 wt.%, mainly with dodecane as the continuous phase. This quantity of particles corresponds to a volume content of 1.8 vol.%. This indicates that the agglomerates of particles are extremely open since a network was obtained at a low silica content into the oily phase. This would confirm that nature of the continuous phase can control the formation and the shape of the network among droplets, built up by the silica particles. This was demonstrated here for liquids with close viscosities.

The analysis of the particles sizes in the continuous phase and at the interface can be briefly discussed. In oil, the size of the particles and aggregates of particles is around 15–30 nm. The size of the particles at the oil/water interfaces is similar to that in the continuous phase. Conversely, the size of the aggregates observed in water range between 10 µm and 400 nm. The interesting feature is that the size of the stabilizer at the interface seems to be around 400 nm or lower. This is due to the presence of the oil in combination with the energy of the stirrer.

### 3.4. Influence of the Viscosity of the Oily Phase

The influence of the viscosity of the oily phase on the organization of the particles in the direct and reverse emulsions is discussed. To this aim, paraffin and dodecane were used since they display a very large difference of viscosity (η_Paraffin oil_/η_Dodecane_ = 100) but have roughly the same chemical structure, in order to have a similar affinity for the silica particles. As previously observed with water and dodecane, it appears that the phase in which the particles are dispersed, i.e., the initial wetting of the particles, impacts the final type of emulsions. Paraffin/W emulsions were obtained when the particles were initially dispersed in water, while W/paraffin emulsions were formed when the silica particles were initially dispersed in the paraffin oil. This aspect has been already reported elsewhere but seems to highlight the versatility of this silica particle.

#### 3.4.1. Direct Oil-in-Water Emulsions

Direct oil-in-water emulsions were obtained when the particles are initially dispersed in the aqueous phase. The droplet size distribution and average droplet diameter are reported in Figure 4b and Table 2.

Paraffin/W emulsions display droplets of around 54 µm while dodecane/W emulsions lead to droplets of a lower diameter of 20 µm. Since the viscosity of the continuous aqueous phase is similar, the size of the droplets is affected by the viscosity of the dispersed phase (η_d_) and also the ratio of viscosities (η_d_/η_c_). While the viscosity of liquid phases was very close for the dodecane–water system, the viscosity of paraffin oil is over 100 times the viscosity of water. Indeed, the dispersed phase with low viscosity resists less to deformation than the high-viscosity dispersed phase. Consequently, the shearing forces produced by a homogenization device deform and break easily the liquid/liquid interfaces with low resistance to deformation (and subsequent breakage).

**Figure 7:**
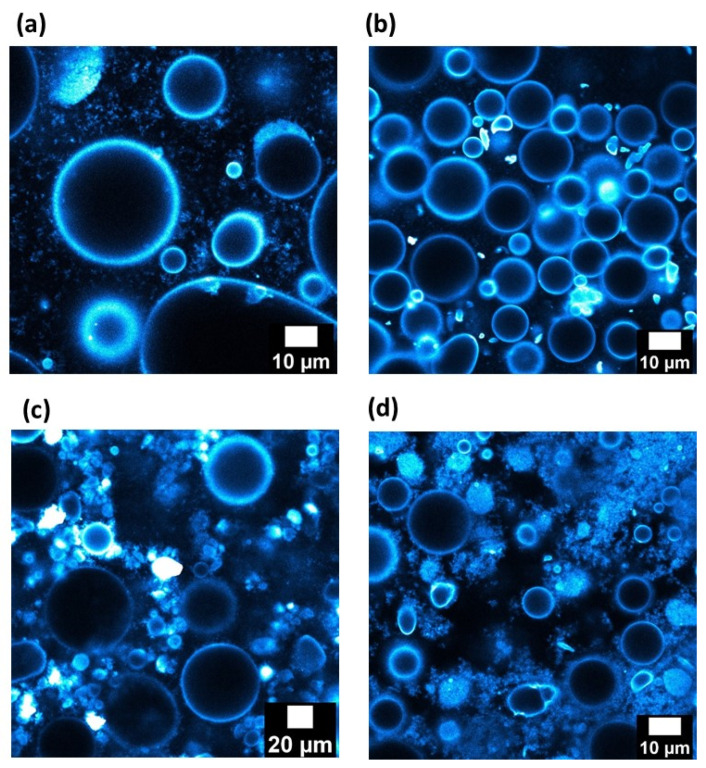
Confocal fluorescence images of (**a**,**c**) Paraffin/W and (**b**,**d**) Dodecane/W direct emulsions prepared with (**a**,**b**) 1 wt.% and (**c**,**d**) 4 wt.% of partially hydrophobic silica particles.

First, the emulsions stabilized by 1 wt.% of silica in the continuous phase were considered. Images taken in fluorescent confocal microscopy are presented in Figure 7a,b, where the silica particles are represented in blue.

**Figure 8:**
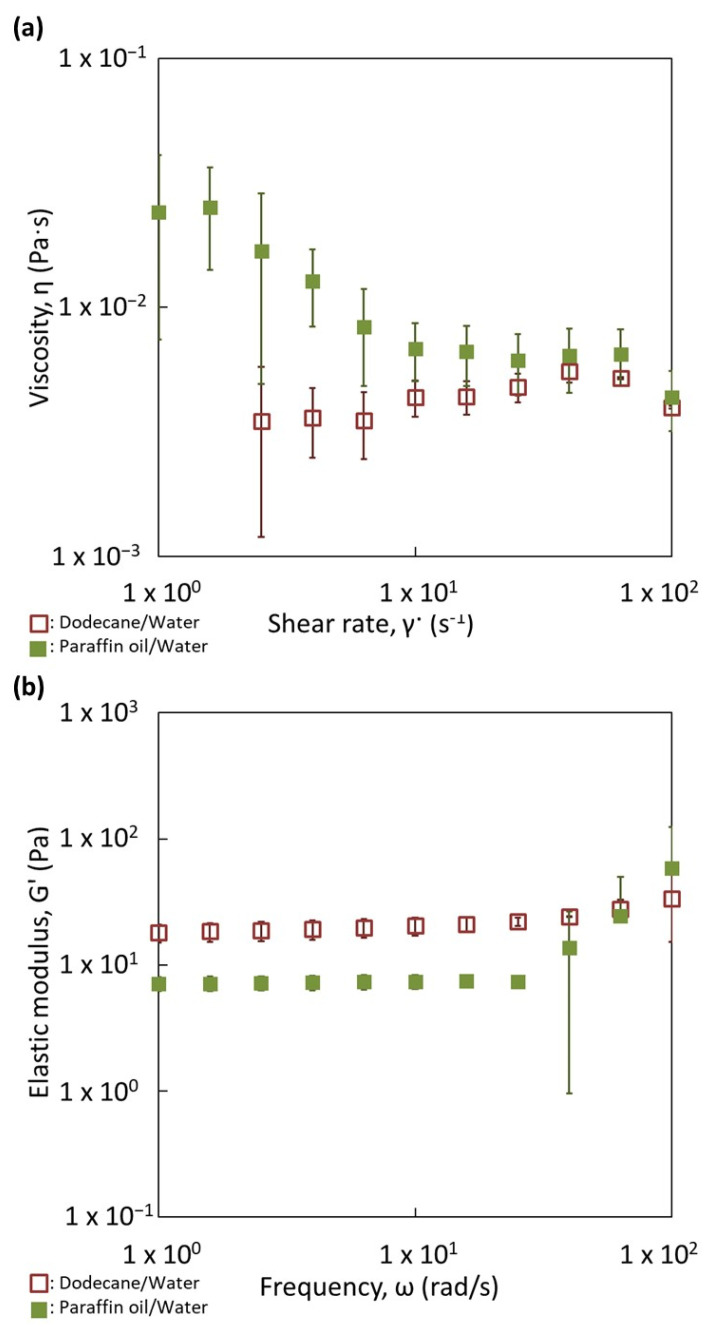
Comparison of the rheological behavior of Paraffin/Water and Dodecane/Water direct emulsions prepared with (**a**) 1 wt.% and (**b**) 4 wt.% of partially hydrophobic silica particles. (**a**) Flow curves. (**b**) Mechanical spectra.

Droplet size distributions of both kinds of emulsions are not the same and they confirm the results displayed in Table 2. The silica particles are adsorbed at the paraffin/water and dodecane/water droplet interfaces, since the droplets have a bluish corona. This suggests that, under our preparation process, the particles have a sufficient time and energy to migrate to the paraffin/water interface, since the particles are initially present inside the water phase. The two emulsions appear rather similar in terms of particle adsorption and organization. For dodecane/W and paraffin/W emulsions, absent or very low amounts of particles were detected in the continuous phase. This is confirmed by the flow curves with the two emulsions, where the viscosity vs. shear rate are rather identical (Figure 8a).

To confirm this trend, which seems to indicate that the viscosity of the oily phase does not impact the organization of the particles for O/W emulsions, the silica content is shifted from 1 wt.% to 4 wt.%. The fluorescent confocal microscopy images of paraffin/w and dodecane/w emulsions are displayed in Figure 7c,d.

The organization of the particles seems not to be substantially affected by the viscosity of the oily phase, since the two pictures are rather similar. In addition, the remaining silica in the continuous phase creates a network with the droplets and the organization of the network does not seem different. To confirm this aspect, the behavior of the elastic moduli as a function of frequency is represented for these two emulsions in Figure 8b. Elastic modulus values for the two emulsions are constant and independent of the frequency until a frequency value of 10 rad/s. This behavior indicates a weak gel behavior of the two systems. More interestingly, the two curves are very close in terms of trends and values. No difference in the organization of the particles can be highlighted. This indicates that the viscosity of the dispersed phase, which does not initially contain the particles, does not affect the repartition of the particles and the network of particles/droplets.

It appears interesting to discuss and summarize the distribution of the particles. At 1 wt.% of silica, the majority of the particles goes to the droplets interfaces. There are almost no particles in the continuous phase. They are either aggregated or at the interface. There are few particles introduced and many interfaces. Consequently, everything is at the interface. In addition, the continuous phase is rather clean, without particles. At 4 wt.% of silica, there are more particles. As the interfaces are saturated with silica, the silica aggregates are found in the continuous phase. These aggregates are similar to those observed in suspensions (Figure 2d).

#### 3.4.2. Reverse Water-in-Oil Emulsions

The impact of the viscosity is expected to play a role in the reverse emulsions, for which the particles are initially placed in the oily phase. Lower droplet sizes were obtained with paraffin rather than with dodecane (Table 3 and Figure 4b). These results confirm that at low-dispersed phase fractions, the shear stress is produced via the continuous phase. Consequently, the shearing is more efficient for the case of high-viscosity liquids.

**Figure 9:**
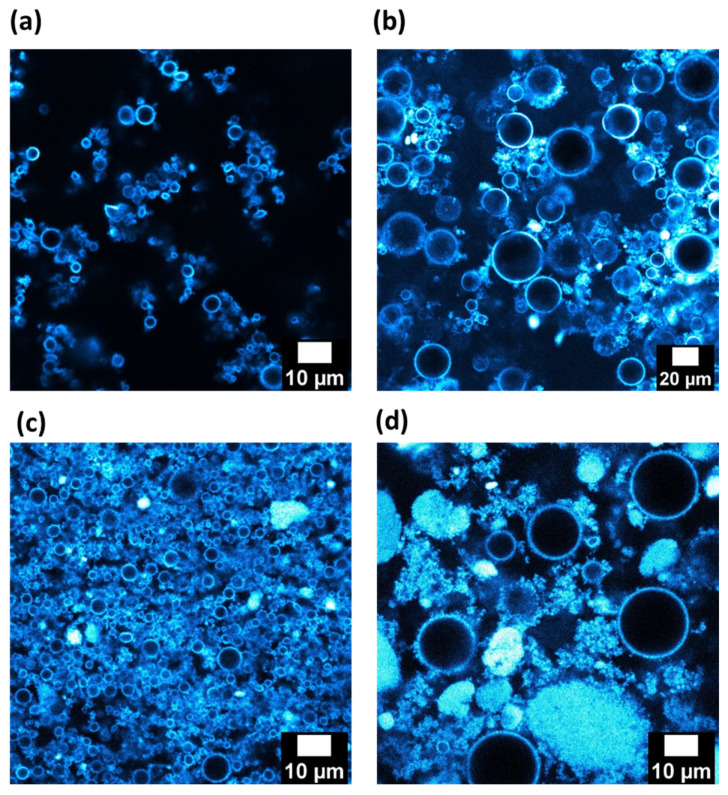
Confocal fluorescence images of (**a**,**c**) Water/Paraffin and (**b**,**d**) Water/Dodecane reverse emulsions prepared with (**a**,**b**) 1 wt.% and (**c**,**d**) 4 wt.% of partially hydrophobic silica particles.

**Figure 10:**
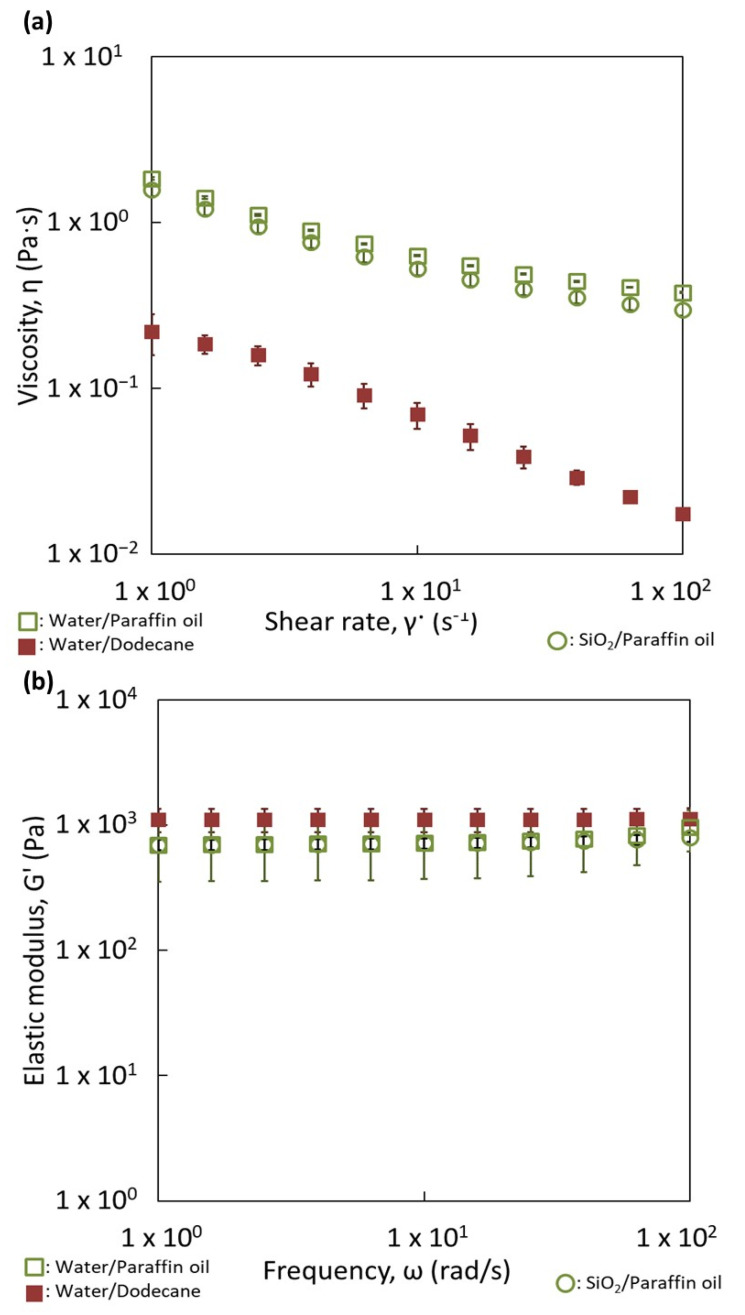
Comparison of the rheological behavior of Water/Paraffin and Water/Dodecane reverse emulsions prepared with (**a**) 1 wt.% and (**b**) 4 wt.% of partially hydrophobic silica particles. (**a**) Flow curves. (**b**) Mechanical spectra.

For the emulsions prepared with 1 wt.% of silica, confocal microscopy (Figure 9a,b) and flow curves (Figure 10a) were used to probe the repartition of the particles. The silica particles are adsorbed at the paraffin/water and dodecane/water interfaces (blue corona around the droplets). This suggests that the silica particles have sufficient time and energy to migrate from the paraffin oily phase to the O/W interfaces despite the higher viscosity of the paraffin oil. In addition, the organization of the particles in the continuous phase appears different. For the W/dodecane emulsion, the particles and the aggregates of the partially hydrophobic silica remain in the continuous phase and they seem to create interconnections among droplets. On the opposite, no aggregates of silica are visible in the paraffin oily continuous phase for W/paraffin emulsions. This can indicate the absence of particles, the presence of well-dispersed particles or a network of individual particles. For the two latter hypothesis, the individual particles are too small to be visible in the picture (Figure 9a). The evidence of the formation of the silica network in the paraffin oil phase should be that W/paraffin emulsions containing silica have similar flow curves to the dispersion of paraffin. The higher viscosity of W/paraffin emulsion compared to that of W/dodecane emulsion attests that the two networks of particles in dodecane and paraffin are different. The experiments with higher silica contents of 4 wt.% are conducted to give new insight on this aspect.

The effect of the continuous phase can be improved by shifting the silica content from 1 wt.% to 4 wt.%. The fluorescent confocal microscopy images of W/paraffin and W/dodecane emulsions stabilized with 4 wt.% of silica are displayed in Figure 9c,d. Distributions of the droplets size correspond fairly well to droplet sizes reported in Table 3. As silica is stained in blue, it is possible to see that all the liquid/liquid interfaces of these emulsions are covered by the particles. This would mean that these silica particles are always capable of moving from the phase where they were originally dispersed (i.e., the continuous phase) to liquid/liquid interfaces, independently of the chemical nature (lipophilic or hydrophilic) and viscosity of the continuous phase. This phenomenon was also observed by Zanini and coworkers, who played on the surface roughness of particles to produce reverse and direct emulsions [12].

For the two emulsions, network of particles/droplets are visible. The networks appear different. This confirms the presence of a network of particles in dodecane and also in paraffin for 1 wt.% of silica. However, less dispersed silica is present in the continuous dodecane phase as compared to those in paraffin. Furthermore, the particles in the emulsions are close to the initial state of aggregation or initial network of silica in the two oily suspensions. However, the dispersion state of the silica particles into the emulsion is not the same whether the continuous phase is viscous or not. A lot of particle aggregates are still present in the dodecane continuous phase, which corresponds to those already observed in a suspension of silica in dodecane (Figure 2b). The presence of aggregates is not obvious when paraffin oil is the continuous phase, which is consistent with the absence of aggregates in the paraffin suspension (Figure 2c). It is interesting to keep in mind that a network of silica/droplets is recorded for the two emulsions. However, the network seems different depending on the viscosity of the oily continuous phase, and consequently, the state of aggregation of the silica in the continuous phase.

The behavior of elastic moduli as a function of frequency is represented for these reverse emulsions in Figure 10b. Elastic modulus values of the reverse emulsions (squares) are constant and independent of the frequency. This confirms the existence of a strong gel for the two emulsions. As already discussed, these high values of G′ and its independency with the frequency are not expected to take place at this low dispersed phase content. They are due to the network of particles in the continuous phase, which link the droplets together. This is probably why the mechanical spectra for water-in-dodecane and water-in-paraffin oil emulsions are fairly close and their values are about 1000 Pa. A similar value can, at a first approximation, indicate that the state of aggregation or interconnection of the silica/droplets is close. The pictures do not confirm this conclusion.

**Figure 11:**
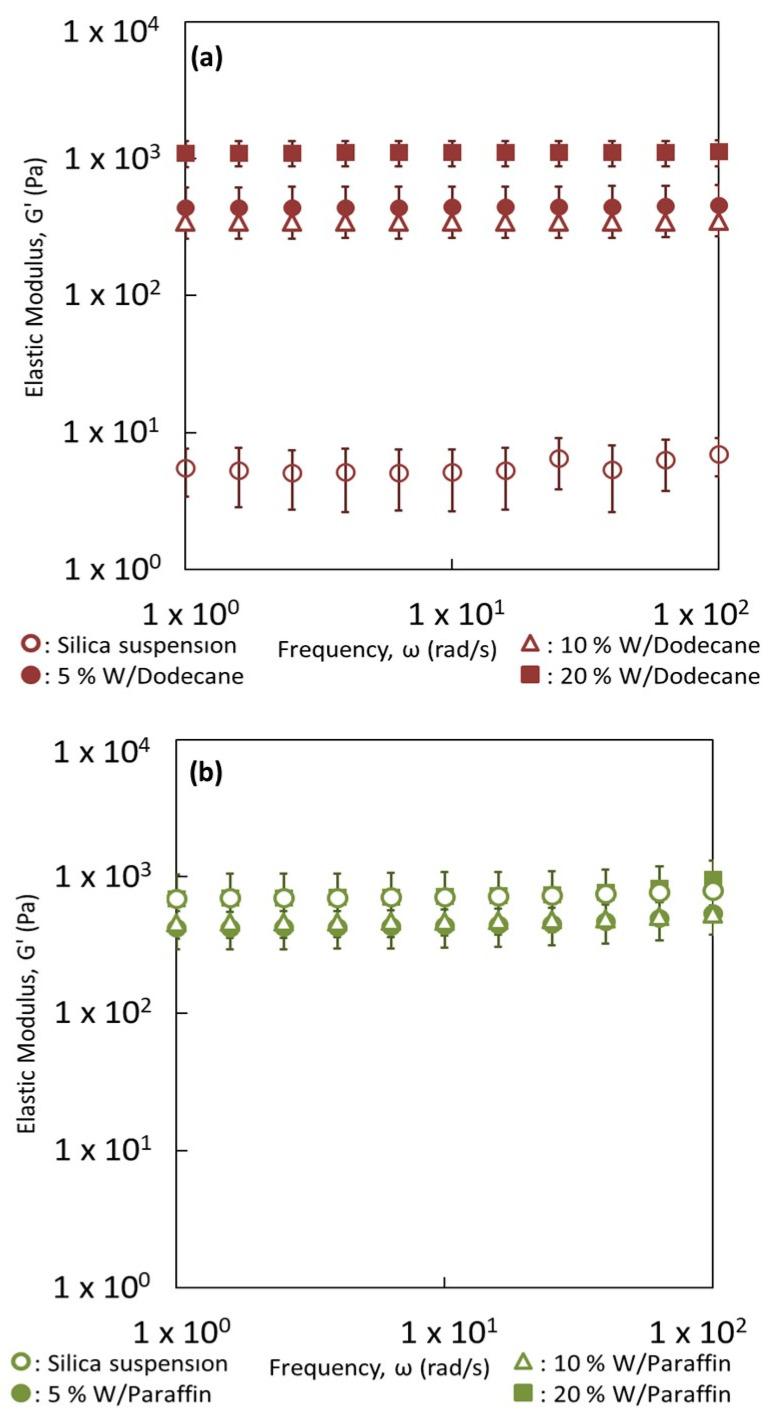
Comparison of the rheological behavior of reverse W/O emulsions with various dispersed phase fractions stabilized with 4 wt.% of silica particles and a suspension of silica particles (4 wt.%) dispersed in (**a**) dodecane and (**b**) paraffin. Dispersed phase fractions of 5%, 10% and 20% are used.

In order to better understand the network formed at large silica content of 4 wt.% for the reverse W/dodecane and W/paraffin emulsions, the evolution of the elastic modulus of the emulsions is followed with dispersed phase fractions lower than or equal to 20%. The results for W/dodecane and W/paraffin emulsions are displayed in Figure 11a,b, respectively.

The evolution of G′ vs. frequency curves with the dispersed phase fractions depends on the viscosity of the oily phase. Two cases can be distinguished depending on the viscosity of the oily phase in which the particles are dispersed. For the water-in-dodecane emulsions, the elastic modulus increases progressively with water addition (Figure 11a). More particularly, a large improvement of G′ occurs from no water to 5% water dispersed phase. Consequently, the rheological behavior is not initially dominated by the silica particles. As the water concentration is increased, the added water droplets contribute to connectivity between the silica particles, leading to an overall increase of the elastic modulus. For the water-in-paraffin oil systems, values of G′ are equal and this is independent of the concentration of the aqueous phase (Figure 11b). The rheological behavior of the system would be dominated by the network of silica particles. The emulsion could be described as a network of silica particles containing water inclusions. In consequence, the viscosity of the oily phase controls the organization of the silica particles. It would allow controlling the manner by which the network among dispersed objects is built up. Surprisingly, the two types of networks reach similar values of G′ at 20% of dispersed phase fraction while for lower dispersed phase fractions, the elastic moduli are different.

#### 3.4.3. Effect of the Initial Wetting of the Particles

From the data with W/paraffin and paraffin/W emulsions, it becomes possible to assess the effect of the initial wetting of the particles, i.e., in paraffin or in water, on the organization of the particles. The data are discussed based on the previous figures (Figure 7, Figure 8, Figure 9 and Figure 10). However, the data are also replotted in Figures S6–S9 of the Supporting Information in order to directly compare the results of W/paraffin and paraffin/W emulsions.

For the direct and reverse emulsions stabilized by 1 wt.% of silica in the continuous phase, the images taken in fluorescent confocal microscopy confirm the different organization of the aggregates of silica when initially dispersed in an oily or aqueous phase (Figure S6 of the Supporting Information). Some agglomerates of about 1–10 µm remain in the aqueous continuous phase, while they are not present in the oily continuous phase. In terms of flow curves, the viscosity of the reverse emulsion appears substantially larger than that of the direct emulsion (Figure S7 of the Supporting Information). Viscosity of the reverse emulsion corresponds to the viscosity of its external phase (continuous phase) with 1 wt.% of silica particles.

For the emulsions stabilized with 4 wt.% of particles, the droplets’ interfaces are also covered by the silica particles (Figure S8 of the Supporting Information). This confirms that the particles can move from the initial phase in which they were introduced (i.e., the continuous phase) to the liquid/liquid interfaces, regardless of the chemical nature (lipophilic or hydrophilic) and the viscosity of the continuous phase. This aspect was reported by other groups [12]. However, the dispersion state of the silica particles into emulsions is not the same whether the continuous phase is oil or water. A lot of particle aggregates are still present in the aqueous continuous phase, which corresponds to those already observed in an aqueous suspension of silica (Figure 2d). The presence of aggregates is not obvious when paraffin oil is the continuous phase, which is consistent with the absence of aggregates in the paraffin suspension (Figure 2c). In terms of rheological properties, the elastic moduli of the reverse emulsion are higher than those of the direct emulsion (Figure S9 of the Supporting Information). These differences are the manifestation of different aggregation states of the silica aggregates, whether dispersed in an oily or aqueous phase. The latter would lead to the most aggregated state of the silica particles, unable to create a strong network of particles. Conversely, a network of particles containing water inclusion occurs for W/paraffin emulsions.

All the data highlight that the main parameter that controls the organization of the particles is the phase in which the particles are initially dispersed.

## 4. Conclusions

The aim of this paper was to address some parameters that can impact the organization of the particles in Pickering emulsions. The influence of the initial wetting of the particles in the oil or in the aqueous phase prior to emulsification, as well as the viscosity of the oil phase on the organization of the particles in the emulsions, was particularly studied. The same partially hydrophobic silica particles (displaying a critical surface energy of 38 mJ/m^2^) were used to stabilize W/O reverse and O/W direct emulsions. Emulsions were prepared with 20 vol.% of liquid dispersed phase and stabilized by 1 and 4 wt.% of silica particles dispersed in an oily or aqueous phase.

The type of emulsion as well as the organization of the particles was significantly affected by the phase in which the particles were initially dispersed. Water and dodecane were used in order to investigate the impact of the viscosity. W/O emulsions were obtained when the particles were initially dispersed in the oily phase, while dodecane/water emulsions were prepared when the silica was initially introduced in the aqueous phase. The silica particles were adsorbed at the liquid/liquid droplet interfaces for all the prepared emulsions. When the particles were initially dispersed in apolar dodecane, the particles remaining in the oily continuous phase created interconnexion among the droplets even at 1 wt.% of silica. Conversely, only a weak amount of particles was present in the aqueous continuous phase when the particles were initially dispersed in water. This particle organization was confirmed and exacerbated in the presence of a larger amount of silica (4 wt.%). In both cases (W/O and O/W emulsions), the remaining silica in the continuous phase created a network with the droplets, but the strength and the organization of the network was different. For W/O emulsions, a strong gel was obtained while a weaker gel was detected in the case of O/W emulsion.

Paraffin oil was also used to evaluate the impact of the viscosity of the oil on the organization of the particles. For dodecane/W and paraffin/W direct emulsions, no difference in the organization of the particles could be highlighted, indicating that the viscosity of the dispersed phase did not affect the repartition of the particles. The impact of the viscosity becomes significant with the reverse emulsions. A network of particles was detected in the continuous phase in both cases. Nevertheless, the networks were very different. For W/dodecane emulsions, the gel behavior was not initially dominated by the silica particles, but the addition of water droplets contributed to promoting the connection between the silica particles. For W/paraffin emulsions, the gel behavior of the system was dominated by the network of particles and the emulsion could be described as a network of silica particles containing water inclusions.

This work highlights that the initial phase in which the particles are dispersed has the major impact on the organization of the particles. The viscosity of the oily phase seems to affect the organization of the particles only for the reverse W/O emulsions. The potential application, in a broad way, is to be able to make direct and reverse emulsions extremely stable with the same stabilizer. The versatility could help in the encapsulation and the controlled release of pharmaceutical active ingredients, both hydrophilic and hydrophobic, with the same particles as a stabilizer.

## Figures and Tables

**Table 1:** Average droplet diameter of reverse W/O and direct O/W emulsions prepared with water and dodecane.

Silica Content (wt.%)	Reverse W/O Emulsions	Direct O/W Emulsions
1	17.7 ± 8.0 µm	19.8 ± 2.6 µm
4	13.9 ± 7.0 µm	19.7 ± 0.8 µm

**Table 2:** Average droplet diameter of direct Paraffin/Water and Dodecane/Water emulsions.

Silica Content (wt.%)	Paraffin/W Emulsions	Dodecane/W Emulsions
1	55.1 ± 12.6 µm	19.8 ± 2.6 µm
4	53.5 ± 1.2 µm	19.7 ± 0.8 µm

**Table 3:** Average droplet diameter of reverse Water/Paraffin and Water/Dodecane emulsions.

Silica Content (wt.%)	W/Paraffin Emulsions	W/Dodecane Emulsions
1	2.3 ± 0.5 µm	17.7 ± 8.0 µm
4	1.9 ± 0.1 µm	13.9 ± 7.0 µm

## Data Availability

Data are contained within the article.

## Supplementary Materials

The following Supporting Information can be downloaded at: https://www.mdpi.com/article/10.3390/nano13030371/s1, Figure S1. Evolution of the elastic (G′) and viscous (G″) modulus of suspension of silica particles dispersed in dodecane as a function of the oscillatory frequency. The symbol δ corresponds to the phase-shift angle. The silica content is equal to 4 wt.%.; Figure S2. Evolution of the elastic (G′) and viscous (G″) modulus of suspension of silica particles dispersed in paraffin as a function of the oscillatory frequency. The symbol *δ* corresponds to the phase-shift angle. The silica content is equal to 4 wt.%.; Figure S3. Evolution of the elastic (G′) and viscous (G″) modulus of suspension of silica particles dispersed in a 2 % brine of NaCl as a function of the oscillatory frequency. The symbol *δ* corresponds to the phase-shift angle. The silica content is equal to 4 wt.%.; Figure S4. Evolution of the elastic (G′) and viscous (G″) modulus of suspension of silica particles dispersed in paraffin as a function of shear strain (γ). The symbol δ corresponds to the phase-shift angle. The silica content is equal to 4 wt.%. Figure S5. Evolution of the elastic (G′) and viscous (G″) modulus of suspension of silica particles dispersed in dodecane as a function of shear strain. The silica content is equal to 4 wt.%. Figure S6. Confocal fluorescence images of (a) reverse and (b) direct emulsions prepared with paraffin oil and 1 wt.% of partially hydrophobic silica particles.; Figure S7. Comparison of the rheological behavior of reverse and direct emulsions prepared with paraffin oil and 1 wt.% of partially hydrophobic silica particles. Flow curves showing the viscosity (η) as a function of the shear rate ($\dot{\gamma }$).; Figure S8. Confocal fluorescence images of (a) reverse and (b) direct emulsions prepared with paraffin oil and 4 wt.% of partially hydrophobic silica particles. Figure S9. Comparison of the rheological behavior of reverse and direct emulsions prepared with paraffin oil and 4 wt.% of partially hydrophobic silica particles. Mechanical spectra showing the elastic modulus (G′) as a function of frequency (ω).
