# Supplementary material for: Particles’ Organization in Direct Oil-in-Water and Reverse Water-in-Oil Pickering Emulsions
Source: Nanomaterials (Basel). 2023 Jan 17;13(3):371. doi: 10.3390/nano13030371 (PMC9919868; doi:10.3390/nano13030371)
Supplement: Supplementary file 1 [file nanomaterials-13-00371-s001.zip › nanomaterials-2026572-Supplementary Materials.pdf]

Supplementary Materials

Particles' Organization in Direct Oil-in-Water and Reverse Water-in-Oil Pickering Emulsions

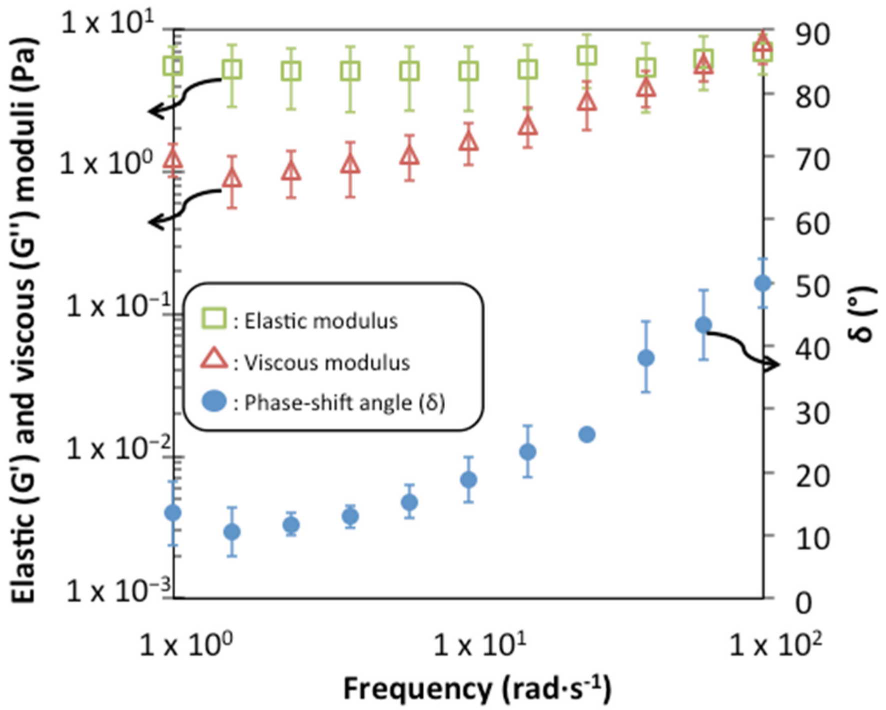

Figure S1. Evolution of the elastic (G') and viscous (G'') modulus of suspension of silica particles dispersed in dodecane as a function of the oscillatory frequency. The symbol δ corresponds to the phase-shift angle. The silica content is equal to 4 wt.%.

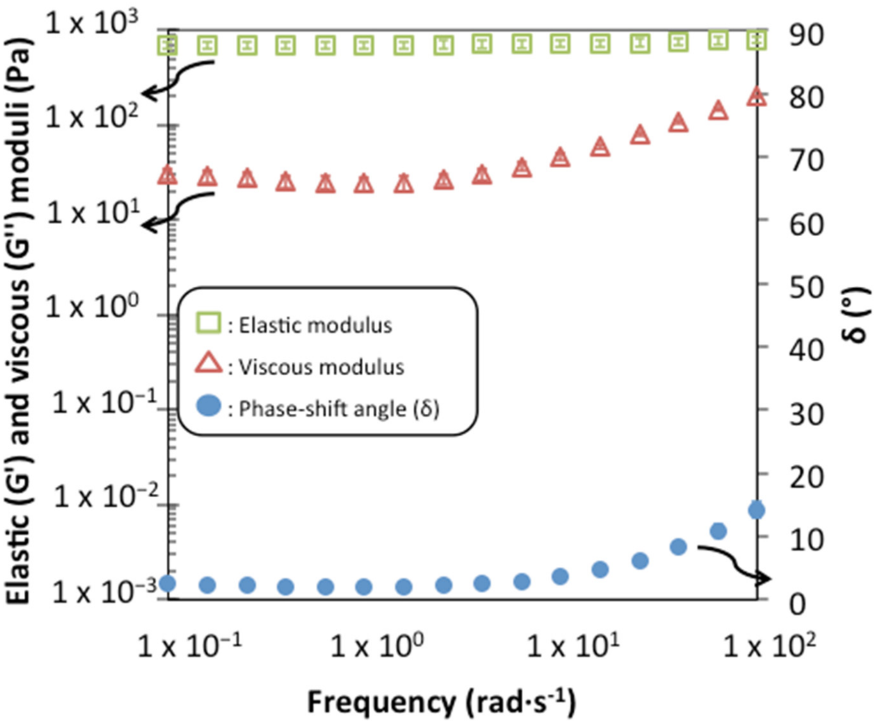

Figure S2. Evolution of the elastic (G') and viscous (G'') modulus of suspension of silica particles dispersed in paraffin as a function of the oscillatory frequency. The symbol δ corresponds to the phase-shift angle. The silica content is equal to 4 wt.%.

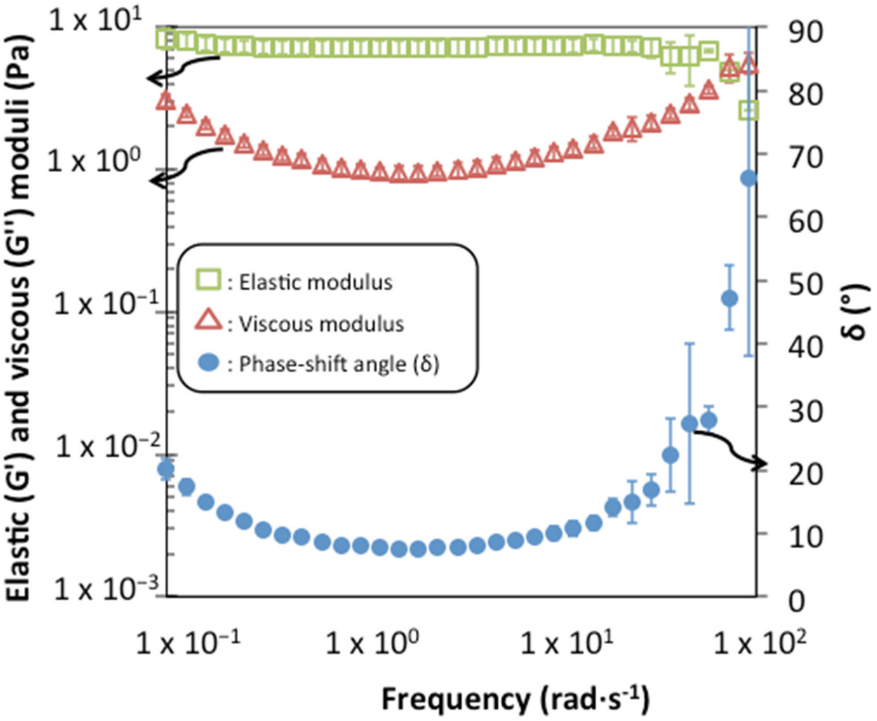

Figure S3. Evolution of the elastic (G') and viscous (G'') modulus of suspension of silica particles dispersed in a 2 % brine of NaCl as a function of the oscillatory frequency. The symbol δ corresponds to the phase-shift angle. The silica content is equal to 4 wt.%.

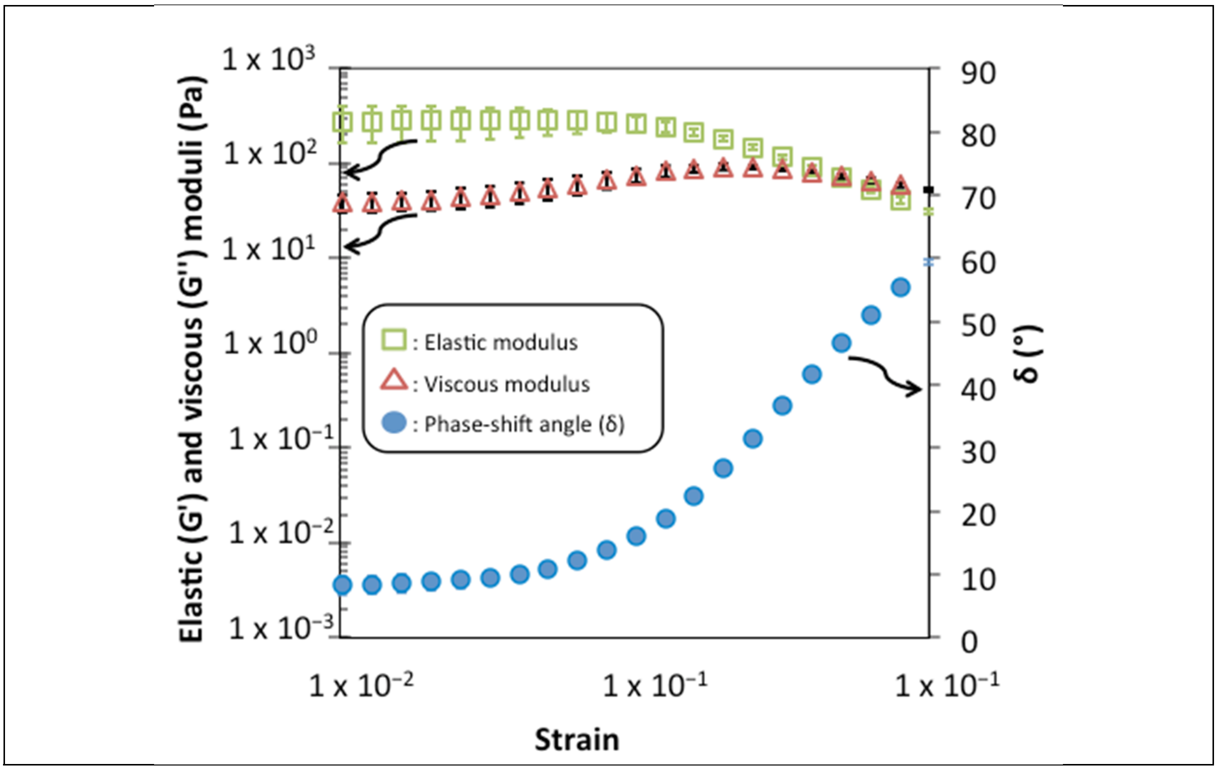

Figure S4. Evolution of the elastic (G') and viscous (G'') modulus of suspension of silica particles dispersed in paraffin as a function of shear strain (γ). The symbol δ corresponds to the phase-shift angle. The silica content is equal to 4 wt.%.

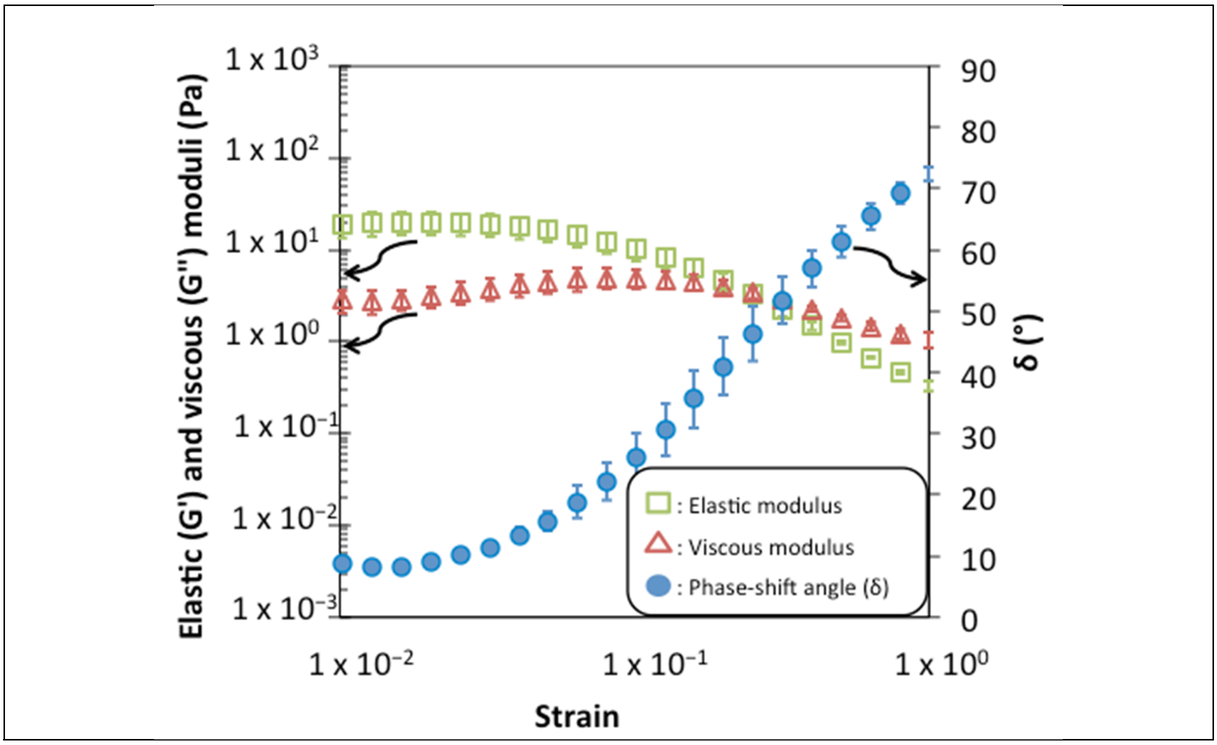

Figure S5. Evolution of the elastic (G') and viscous (G'') modulus of suspension of silica particles dispersed in dodecane as a function of shear strain. The silica content is equal to 4 wt.%.

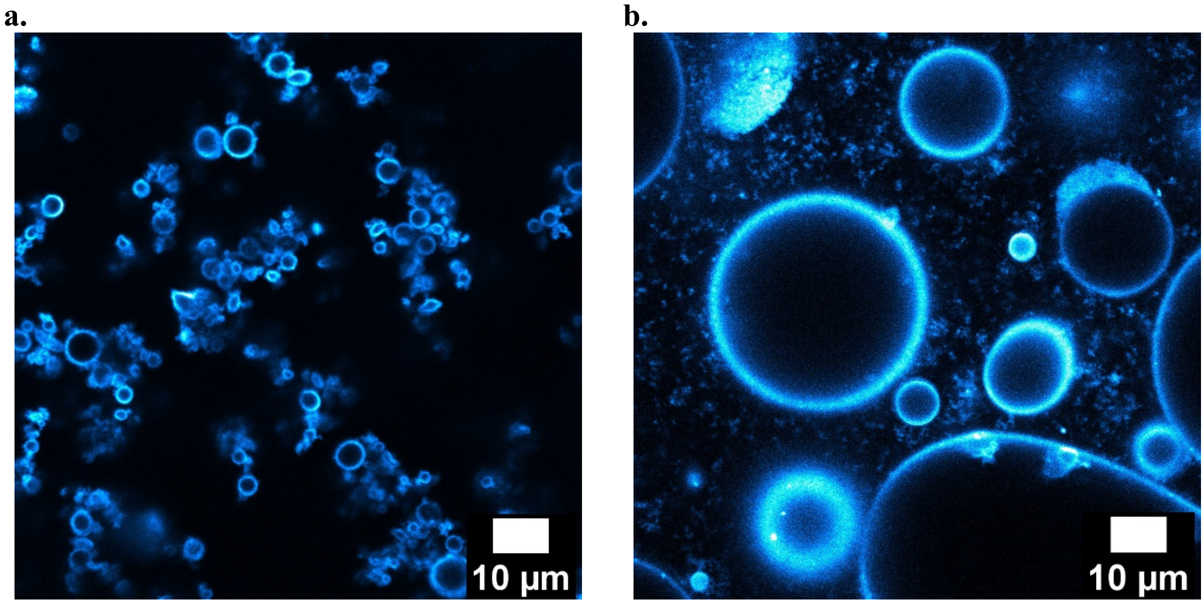

Figure S6. Confocal fluorescence images of (a) reverse and (b) direct emulsions prepared with paraffin oil and 1 wt.% of partially hydrophobic silica particles.

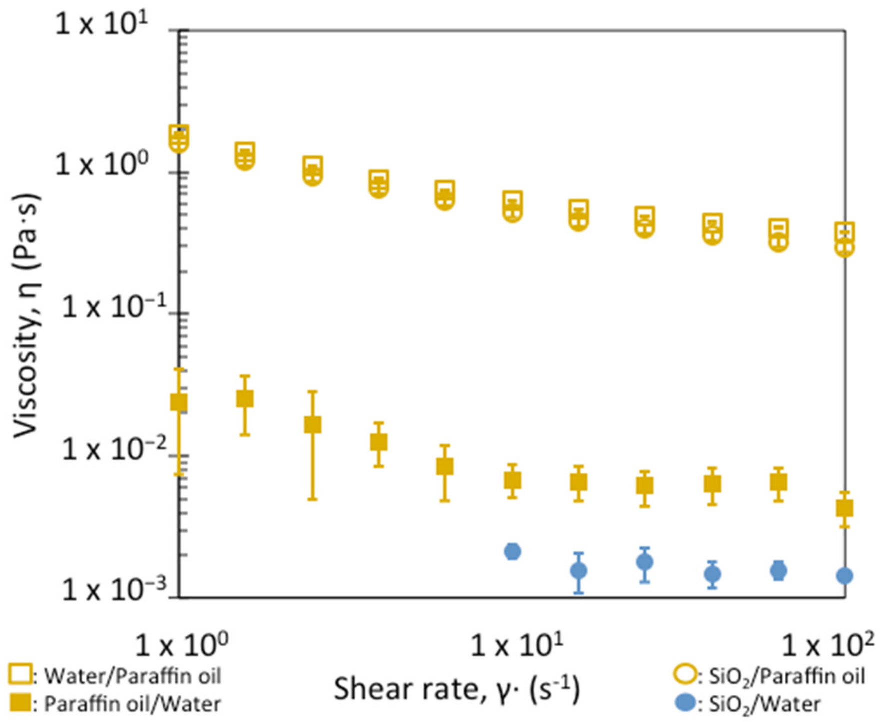

Figure S7. Comparison of the rheological behavior of reverse and direct emulsions prepared with paraffin oil and 1 wt.% of partially hydrophobic silica particles. Flow curves showing the viscosity (η) as a function of the shear rate ($\dot{\gamma}$).

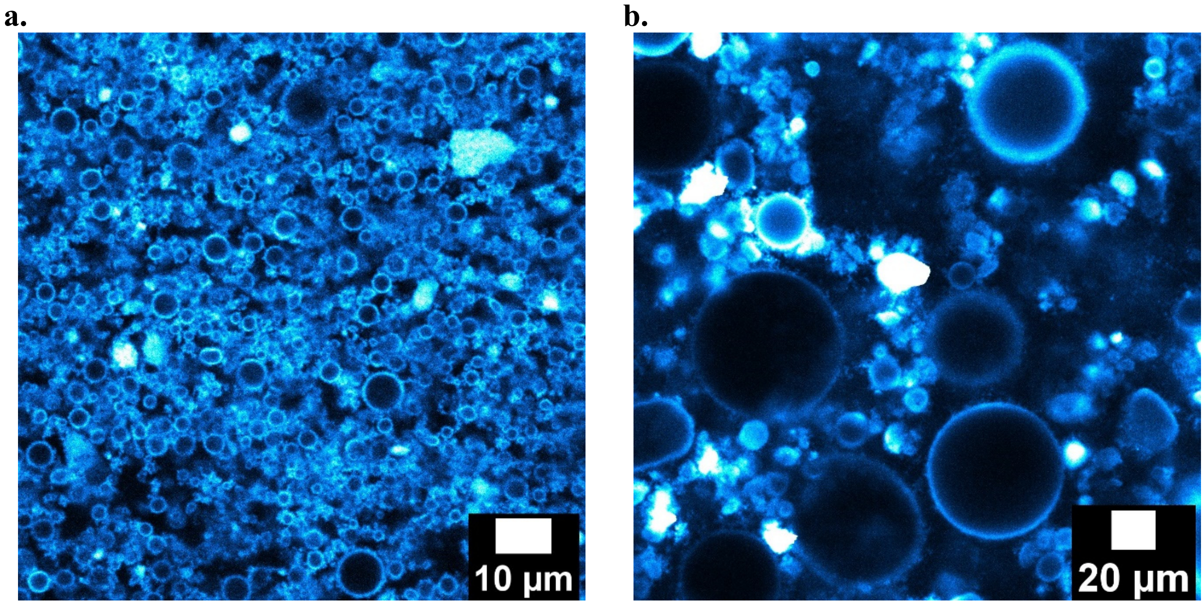

Figure S8. Confocal fluorescence images of (a) reverse and (b) direct emulsions prepared with paraffin oil and 4 wt.% of partially hydrophobic silica particles.

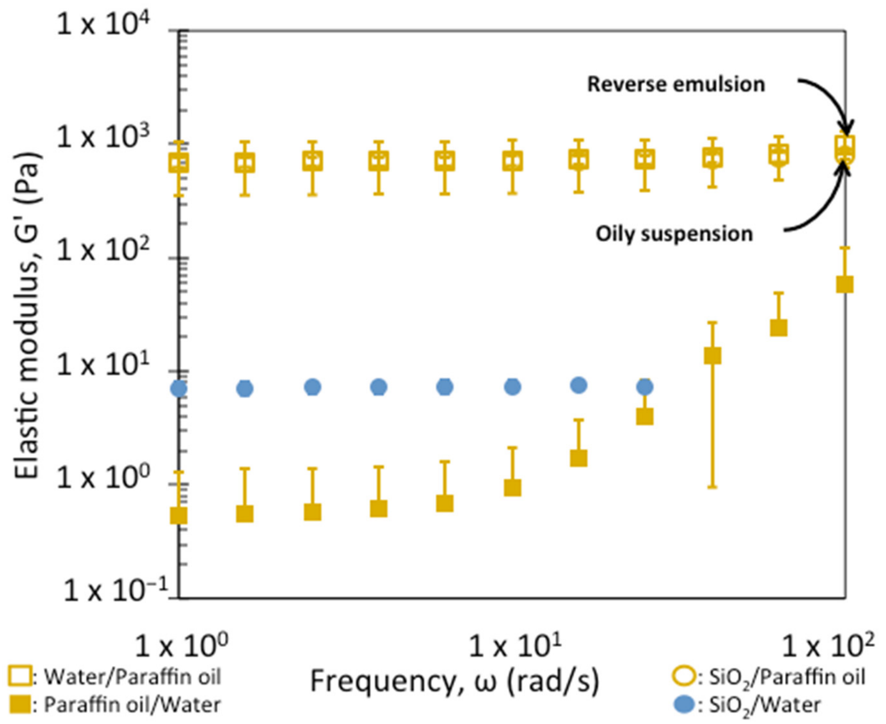

Figure S9. Comparison of the rheological behavior of reverse and direct emulsions prepared with paraffin oil and 4 wt.% of partially hydrophobic silica particles. Mechanical spectra showing the elastic modulus (G') as a function of frequency (ω).
